# THE POWER OF LOOKING AHEAD? A FIXED-EFFECTS MODEL OF FUTURE ASPIRATIONS OVER THE LIFE COURSE AND INCOME

**DOI:** 10.1093/geroni/igz038.018

**Published:** 2019-11-08

**Authors:** Ioana Sendroiu, Laura Upenieks

**Affiliations:** University of Toronto, Toronto, Canada, Canada

## Abstract

Perceived life trajectories are rooted in structural systems of advantage and disadvantage, but individuals also shape their futures through setting goals and expectations. “Future aspirations” have typically been used in life course research to refer to one’s conception of their chances of success across life domains and can serve as a resource to help individuals persevere in the face of hardship. Taking a life course approach and using three waves of data from the MIDUS study, we utilize hybrid fixed effects models to assess the relationship between future aspirations and income. We find that, net of age, health, and a host of other time-varying factors, more positive future aspirations are indeed related to higher income over time, but that this relationship takes different shapes in different contexts. In particular, in lower quality neighborhoods, higher future aspirations lead to worse economic outcomes over the life course, while in higher quality neighborhoods, higher aspirations are indeed related to higher incomes. We thus argue that aspirations are only helpful in some contexts, and are inherently contextual not just in their sources but also in their effects.

